# Well‐being and COVID‐19‐related worries of German children and adolescents: A longitudinal study from pre‐COVID to the end of lockdown in Spring 2020

**DOI:** 10.1111/jcv2.12004

**Published:** 2021-03-17

**Authors:** Mandy Vogel, Christof Meigen, Carolin Sobek, Peggy Ober, Ulrike Igel, Antje Körner, Wieland Kiess, Tanja Poulain

**Affiliations:** ^1^ LIFE Child Leipzig University Hospital for Children and Adolescents Leipzig University Leipzig Germany; ^2^ Department of Women and Child Health Center for Pediatric Research Leipzig University Leipzig Germany; ^3^ Center for Research and Transfer (FTZ) at the Leipzig University of Applied Sciences (HTWK) Research Field Health and Social Affairs Leipzig Germany

**Keywords:** COVID‐19, wellbeing, worries, children, adolescents

## Abstract

**Background:**

There is concern that pandemic measures put a strain on the health and well‐being of children. We investigated the effects of the COVID‐19 pandemic, the lockdown, and social distancing on the well‐being, media use, and emotions of children and adolescents between 9 and 18 years.

**Methods:**

We used linear and proportional odds logistic regression correcting for age, sex, and socioeconomic status (SES) and to compare media use, peers/social support, physical, and psychological well‐being between 2019 (pre‐COVID baseline) and two time points shortly after the start of the lockdown (last week of March and April 2020, respectively) in 391 9–19‐year‐old healthy children and adolescents of the LIFE Child cohort. COVID‐19‐related feelings and their relationship to age, sex, and SES were assessed at two time points during lockdown.

**Results:**

We found significantly lower scores in physical and psychological well‐being during lockdown compared to baseline. The effect was significantly stronger in children with medium/low SES. Perceived social support scores were also significantly lower during the lockdown. The percentage of children who had no contact with their peers (in‐person or online) increased from 3% pre‐COVID to 14% and 13% in April and March 2020, respectively. About 80% of the children missed in‐person contacts with friends. Most of the children worried more about the health of their families than their own. Sixty percent worried about the international situation at least moderately, whereas only 20% were afraid of COVID‐19 itself. The percentage of children who believed it would never be as before COVID‐19 rose from 7.4% at the beginning lockdown end of March to 16.2% a month later. In contrast, all other COVID‐19‐related worries, showed a (nonsignificant) decline during the same period.

**Conclusion:**

Our study supports the notion that pandemic measures have to be balanced against adverse public health effects. Especially vulnerable groups have to be protected.

## Graphical abstract

Our study found a decreasing psychological and physiological wellbeing and an increasing proportion of schoolchildren with no contact with friends and peers during the first COVID‐19‐lockdown. In addition, we observed higher media use and a loss of daily structure.

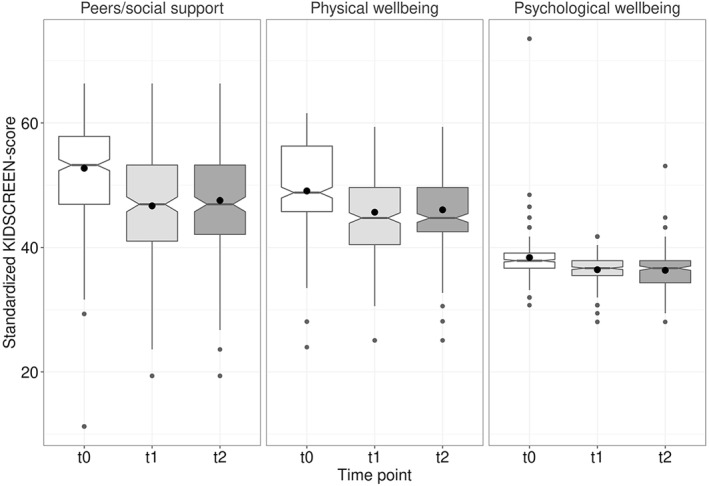

## 1 INTRODUCTION

Children benefit from daily routine and social engagement (Weaver & Wiener, 2020); both are cut short during an epidemic. On March 11, 2020, the World Health Organization labeled COVID‐19 a pandemic. The spread of COVID‐19, caused by the severe acute respiratory syndrome coronavirus 2, reached Germany at the end of January 2020. In mid‐February, multiple cases related to holiday trips and the carnival led to a rising number of COVID‐19 cases. The first schools and kindergartens were closed on March 5. Yet, on March 6 and 9, the German Health Minister ruled out a general closure of kindergartens and schools. Only 9 days later, on March 18, schools and kindergartens were closed all over Germany. Like these closures, the following lockdown and the social distancing rules came for most of the population unexpectedly.

**Key points**

- We found that the physical and mental wellbeing in children and adolescents was significantly lower during the lockdown than during 2019 before the pandemic.
- We were able to compare pandemic data against a prepandemic baseline looking at things from the child's point of view.
- Implication: Measures against a pandemic have to be balanced against adverse public health effects, especially vulnerable groups like children and adolescents must be protected as they are more susceptible to the negative consequences.

COVID‐19 has a large impact worldwide. It has caused an economic decline, panic buying, a rising fear of recession, and a new wave of xenophobia and racism (Agarwal & Sunitha, 2020). Worldwide, school closings affected more than 500 million students (Agarwal & Sunitha, 2020), disrupting their lives (Golberstein et al., 2020), and cutting short social support (Winter et al., 2020). Isolation and uncertainty are difficult to stand for people of all ages (Wagner, 2020) but even more so for children who never experienced a similar situation. Although children seem to be less affected by COVID‐19 symptoms, they lived through a phase of rapid changes in their daily routines and experienced a loss of self‐determination. Their parents may experience a loss of income due to childcare requirements or workplace closures (Douglas et al., 2020). The anticipated economic crisis will likely cause an increase in mental health conditions and addiction problems. Moreover, public health specialists apprehend an increase in unhealthy lifestyle behaviors (Wang et al., 2020). The ramifications on daily life may include a lack of physical activity (PA), higher media use, and a higher duration of sedentary activities. Physical activity is crucial for the physiological, psychological, and social health of children and adolescents (Poitras et al., 2016). Lower levels of PA often accompanied by higher media use/screen time are associated with a higher prevalence of overweight and obesity (Keane et al., 2017; Robinson et al., 2017), unfavorable body composition, higher metabolic risk, lower fitness, lower self‐esteem, and lower prosocial behavior (Carson et al., 2016). Previous studies have shown that a lack of daily structure, for example, during holidays, may be accompanied by an increase in screen time and a decrease in PA (Wang et al., 2020). Similar effects were observed during the COVID‐19‐related lockdown in Spring 2020. Xiang et al. (2020) examined PA and screen time in more than 2400 Chinese children aged 6–17 years. They found a tremendous increase in screen time of 30 h/week and inactivity from 21.3% to 65.6%. Pietrobelli et al. (2020) reported similar effects in 41 obese 6–18‐year‐old children in Italy. These results are supported by Dunton et al. (2020), Zheng (2020), and Moore et al. (2020) who showed a high increase in sedentary leisure activities and lower levels of PAs in children, adolescents, and young adults approximately 1 month into the pandemic in the United States, Canada, and Hong Kong.

Besides the decrease of PA, social isolation is a key threat to the health and well‐being during COVID‐19. The review by Loades et al. (2020) found evidence of increased mental health problems, especially related to loneliness and social isolation in children and adolescents. Loneliness is associated with lower quality of life and higher perceived stress (Mikkelsen et al., 2020), higher prevalence of depression (Goosby et al., 2013; C. H. Liu, Zhang et al., 2020), anxiety (C. H. Liu, Zhang et al., 2020), suicidality (Shovestul et al., 2020), and somatic symptoms (C. H. Liu, Zhang et al., 2020; S. Liu, Liu et al., 2020). Moreover, Shovestul et al. (2020) shows that adolescents and young adults are disproportionately often affected by perceived loneliness.

In the context of COVID‐19, Xie et al. (2020) reported higher prevalence of anxiety and depression symptoms in 2330 primary school students 1 month after the lockdown started. Like‐wise, a study in young adults in the United States found that social distancing and COVID‐19‐related worries were associated with higher levels of anxiety, depression, and symptoms of posttraumatic stress syndrome (C. H. Liu, Zhang et al., 2020; Zhou et al., 2020). A British study revealed a positive correlation of age and anxiety about economic consequences of the COVID‐19 pandemic and between the occurrence of health problems and anxiety about being infected in 698 adolescents (McElroy et al., 2020).

Unlike other disasters, a pandemic's response strategy does not include places where affected individuals find the support they need (Sprang & Silman, 2013) but requires social distancing. The lack of social support, combined with the unpredictability of the situation, might cause adverse long‐term effects. Therefore, our study aimed to examine COVID‐19‐related fears and changes in wellbeing and media use in 9–18‐year‐old children and adolescents caused by the lockdown and social distancing in Germany. We hypothesized an increased media use and a decline in well‐being during the COVID‐19 lockdown compared to before the pandemic. Besides, we examined whether these changes depend on age, sex, and socioeconomic status (SES).

## 2 METHODS

### 2.1 Study design and population

The data were collected within the LIFE Child study, an ongoing childhood cohort study. LIFE Child is conducted at the LIFE Research Center for Civilization Diseases at the University of Leipzig, Germany. It aims to describe the healthy development of children and to identify risk and resilience factors for the development of lifestyle diseases (Poulain et al., 2017). Children are nonrandomly recruited via different institutions like outpatient clinics, kindergartens and schools, and media advertising (radio, TV, Internet, public transportation) (Poulain et al., 2017). Data for the current substudy was collected between March and May 2020. The 9–18‐year‐old participants live in Leipzig and its surroundings, a region only mildly affected by COVID‐19. The LIFE Child study was approved by the Ethical Committee (Institutional Review Board [IRB]) of the Medical Faculty, University of Leipzig, and is registered in the clinical trials database (NCT02550236). The Ethical Committee is registered as an IRB with the Office for Human Research Protection (IORG0001320 and IRB00001750).

### 2.2 Data

Data were collected at three time points, at the most recent regular study visit before the pandemic (t0, mean difference to t1: 0.75 years), at the beginning of school closures (t1), and 1 month later (t2). A total of 608 children between 9.5 and 18.99 years of age were invited to answer the online questionnaires during the week following March 24 (t1) and April 24 (t2) via email.

In the present study, the children's well‐being, feelings regarding the pandemic, media use (all child‐reported), and the pandemic's impact on the parents' working situation (parent‐reported) were assessed.

To assess children's well‐being within the last week, three scales from the KIDSCREEN‐27 questionnaire were used: physical well‐being, psychological well‐being, and peers and social support. Since personal contact was prohibited during the survey period, we added the words “including online and via phone” to the question of whether the children spent time with their friends. The scales were *t*‐standardized according to the KIDSCREEN manual (Ravens‐Sieberer et al., 2007). The other questions of the child questionnaire analyzed here were:

A) When you think of the dangers of Corona and the limitations of countermeasures, how much do you worry? (answer categories: not at all, a little, moderately, rather, completely): 1. About yourself. 2. Your family. 3. Your friends and peers. 4. The situation in your hometown. 5. The situation in Germany. 6. The situation in other countries/the entire world.
B) How much do you agree with the following statements? (answer categories: not at all, a little, moderately, rather, completely): 1. I am afraid of Corona. 2. I don't care about Corona. 3. The precautions are exaggerated. 4. I inform myself regularly about the current developments in the Corona crisis. 5. I suffer from not seeing my peers. 6. I'd rather go back to school normally (only t2).
C) How soon do you think the situation will be back to normal? (answer categories: in 2 weeks, in 1 month, in half a year, it will never be the same as before the Corona crisis).
D) On average, how long did you spend each day in the last week doing the following things? (answer categories: >4 h, 3–4 h, 1–2 h, about 30 min, not at all): 1. Watching films/series via TV, DVD, streaming, etc. 2. Computer games. 3. Social media—active communication. 4. Social media—watching, reading, etc. 5. Reading—books/E‐books. 6. Reading—magazines online and on paper. 7. Listen to music/radio. At t1, this question was asked separately for weekdays and weekend.

Of the questions answered by parents, the question on whether their job situation was negatively affected by the COVID‐19 crisis (either through fewer working hours, subsidized temporary layoff, or loss of job) was included in the present analyses.

These data were joined to the data collected before the pandemic during the last visit to our study center (t0). Data on child's well‐being (KIDSCREEN), media use, and family's SES could be added for 95% of the children. The family SES was given as a combination of education and professional qualification of the parents and the equivalized disposable household income. The respective score ranges from 3 to 21 and was classified into “low,” “medium,” and “high” according to cut‐off values from a German norm sample (Lampert et al., 2014). Because low SES was heavily underrepresented in our sample, “low” and “medium” were combined into one group (“medium/low”).

### 2.3 Statistical analysis

Descriptive statistics are given as mean and standard deviation for continuous variables and counts and percentages for discrete variables (Table 1). KIDSCREEN scores at t0, t1, and t2 were compared using paired and unpaired *t*‐tests. Proportions were compared using *χ* ^2^ and proportion tests. Regression models were applied to test associations between the outcomes and putative covariates (age, sex, SES, negatively affected job situation of the parents). Linear regression was applied for continuous outcomes (change in the KIDSCREEN scores) and proportional odds logistic regression (polr) for ordinal outcomes (Questions A, B, C; differences in media use [Question D] between weekdays and weekend and between t0 and t1). First, univariate regression analyses were performed. Subsequently, associations were adjusted for age and sex (Model 1), and SES (Model 2). *p* Values resulting from polr were determined using normal approximation. The respective effects were reported as odds ratios (OR). Differences in effects were tested between age groups (preteens [9 to <13 years], adolescents [13 to <16 years], and emerging adults [16–18 years]) and sexes. The significance level was set to *α* = 0.05. *p* Values were adjusted for multiple testing using the method by Benjamini and Hochberg (1995).

**TABLE 1:** Characteristics of the study sample

	Male	Female	*p* Overall
	*n* = 195	*n* = 196	
Age t0 (years)	12.4 (2.40)	12.7 (2.54)	0.340
Age t1 (years)	13.1 (2.42)	13.4 (2.50)	0.206
Participants at t1	154 (79.0%)	167 (85%)	0.140
Participants at t2	127 (65.1%)	132 (67%)	0.721
Time difference t0‐ >t1 (years)	0.72 (0.29)	0.78 (0.34)	0.067
Socioeconomic status			0.649
Low	4 (2%)	2 (1%)	
Middle	102 [52%]	100 (51%)	
High	85 (44%)	92 (47%)	
Missing	4 (2%)	2 (1%)	
COVID‐19 among friends/family t1			0.422
No	155 (79.5%)	156 (80%)	
Yes, but only mild symptoms	7 (3.59%)	12 (6%)	
Missing	33 (16.9%)	28 (14%)	
COVID‐19 among friends/family t2			0.200
No	130 (66.7%)	123 (63%)	
Yes, but only mild symptoms	9 (4.62%)	19 (10%)	
Yes, at least someone is seriously ill	2 (1.03%)	4 (2%)	
Missing	54 (27.7%)	50 (25%)	

*Note*: Summary statistics are given as mean and standard deviation for continuous variables and counts and percentages for discrete variables. The respective tests for differences between males and females were *t*‐tests and *χ* ^2^ tests.

## 3 RESULTS

The questionnaires were sent to 608 children aged between 9 and 18 years 317 children answered the questions at t1 and 257 at t2, and 187 completed the questionnaires at both time points. At t1/t2, the response rates were 52%/42%, respectively. Overall, 64% of the children responded at least once. Children did not differ in any variable dependent on participation only at t1, only at t2, or at both t1 and t2 (Table S1). Most effects occured from t0 to t1. Only few variables changed between t1 and t2.

### 3.1 Well‐being

**FIGURE 1:**
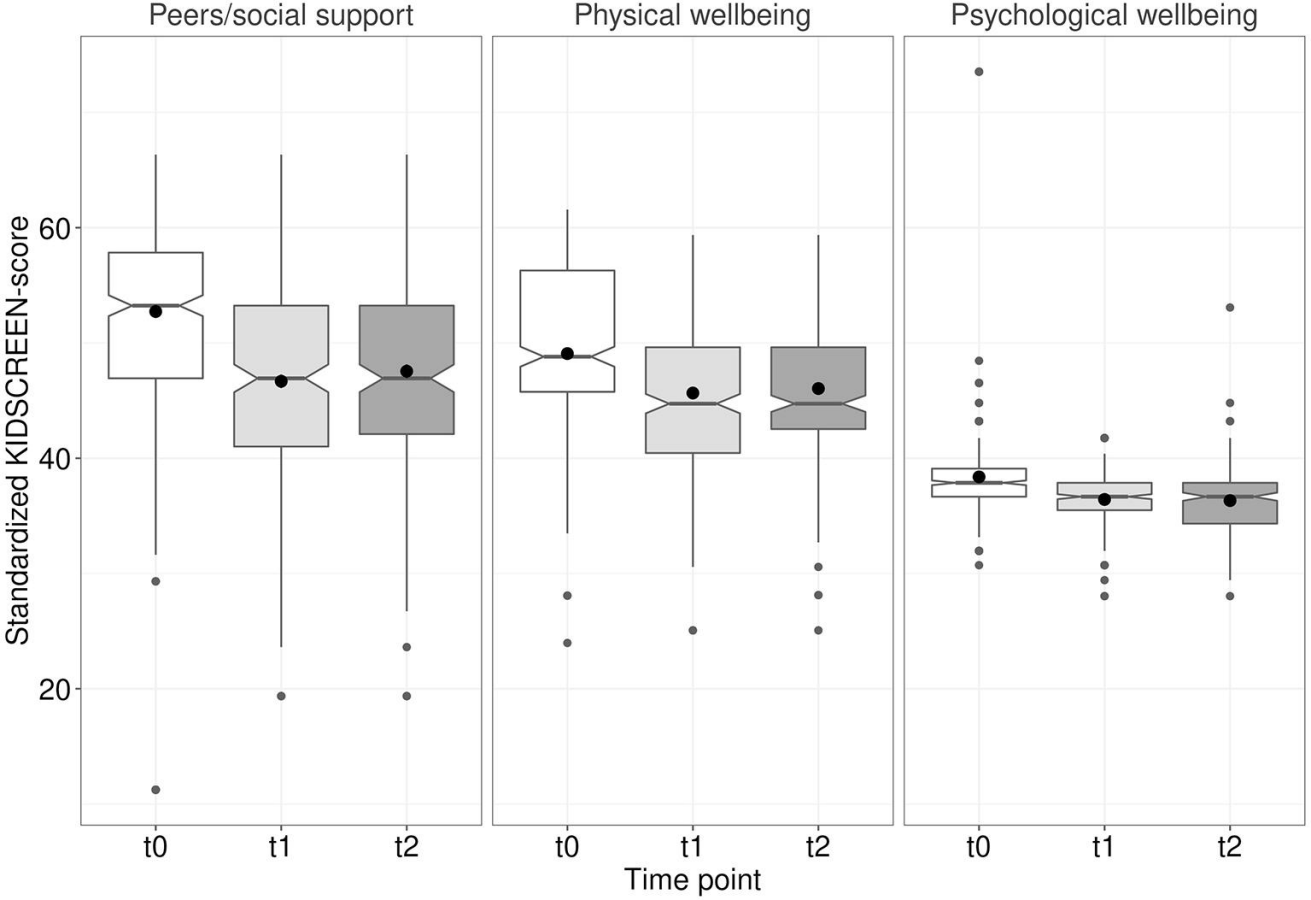
KIDSCREEN scores at t0 (before COVID‐19), t1 (end of March 2020, start of the lockdown), and t2 (end of April 2020): Children showed significantly lower scores at t1 and t2 than before the COVID‐19 crisis

The KIDSCREEN scores were significantly lower at t1 and t2 than at t0 before COVID‐19 (*p* < 0.001 for all scales; Table S2). After adjusting for age and sex, the changes from t0 to t1 were related to the family's SES (physical well‐being: *β*_medium/low_ = −1.6 [*p* = 0.191]; psychological well‐being: *β*_medium/low_ = −1.3 [*p* = 0.144], peers and social support: *β*_medium/low_ = −2.0 [*p* = 0.323]), that is, the decrease was more pronounced in the medium/low socioeconomic subgroup (Table 2). However, after adjusting for multiple testing none of the effects were significant anymore. Only the change in the peers/social support scale was significantly related to age (*β* = 1.3, *p* < 0.001) with milder effects for higher age. Accordingly, the effect was highest in the youngest age group (*β* = −9.5, *p* < 0.001). For all three KIDSCREEN scales, there was no significant difference between t1 and t2 (Table S2 and Figure 1). Although we included phone/online contacts explicitly, the proportion of children who spent very little or no time at all with their friends was around three times as high at t1 (29%) and t2 (28%) as at t0 (10%, *p* < 0.001), indicating an increase of isolation (Table 2)).

**TABLE 2:** Associations of the decrease from t0 to t1 in the three KIDSCREEN scales with age, sex, and SES

Outcomes differences in	Covariates	Analyses					
		Univariate		Age/sex‐adjusted		SES‐adjusted	
		*β*	*p* Value	*β*	*p* Value	*β*	*p* Value
Physical well‐being	SES mid/low (vs. high)	−1.62	0.191	−1.62	0.191		
Peers/social support	SES mid/low (vs. high)	−1.65	0.444	−2.04	0.323		
Psychological well‐being	SES mid/low (vs. high)	−1.31	0.144	−1.35	0.140		
Physical well‐being	age_ch	−0.06	0.909	−0.09	0.795	−0.02	0.913
Peers/social support	age_ch	1.28	<0.001	1.28	*p* < 0.001	1.21	<0.001
Psychological well‐being	age_ch	0.10	0.650	0.10	0.650	0.11	0.437
Physical well‐being	sex_chfemale	1.72	0.157	1.76	0.147	1.81	0.144
Peers/social support	sex_chfemale	0.06	0.973	−0.17	0.973	0.06	0.993
Psychological well‐being	sex_chfemale	0.04	0.993	0.00	0.996	0.21	0.912

Abbreviation: SES, socioeconomic status.

### 3.2 COVID‐19‐virus related worries

Regarding the questions of how much children worry about themselves, their families, their hometown, their country, and the entire world (Question A), the children were least concerned about themselves. Around 67%/70% were “not at all” or just “a little” concerned at t1/t2. They worried most about their families; approximately 85% were at least “moderately” concerned at t1 and t2 (Table 3). There was a tendency of fewer worries at t2 compared to t1. Proportional odds logistic regression revealed odd ratios between 0.68 and 0.94, but only the effect for “worry about the home town” reached the level of significance (OR = 0.68, *p* = 0.041). Children of whom at least one parent's job situation was negatively affected by pandemic were significantly more concerned about their family (OR = 1.49, *p* = 0.010) but less concerned about the international situation (OR = 0.64, *p* = 0.010). Medium/low SES was related to more worries about oneself, the family, and peers/friends but less worry about hometown, country, and the world. However, none of the effects reached statistical significance. Higher age was related to less worry about oneself (OR = 0.86, *p* < 0.001), family (OR = 0.91, *p* = 0.010), peers/friends (OR = 0.87, *p* < 0.001), and the hometown (OR = 0.89, *p* = 0.001). Girls worried significantly more about the country (OR = 1.72, *p* = 0.003) and the world (OR = 1.67, *p* = 0.005) than boys. A similar pattern was present in worries about friends (OR = 1.45, *p* = 0.056) and the hometown (OR = 1.41, *p* = 0.093).

**TABLE 3:** Distribution of answers to the COVID‐19 related questions (*n*_t1_ = 321, *n*_t2_ = 259)

	Worry about											
	Myself (%)		Family (%)		Friends (%)		Hometown (%)		Germany (%)		World (%)	
	t1	t2	t1	t2	t1	t2	t1	t2	t1	t2	t1	t2
Not at all	34.6	35.6	3.0	5.3	17.8	19.0	19.1	29.1	12.1	15.8	11.4	15.4
A little	32.2	34.4	16.1	18.6	33.2	35.6	37.2	33.6	28.9	32.8	23.5	26.3
Moderately	19.5	18.2	25.2	29.1	27.2	22.7	27.2	25.9	33.2	29.6	30.2	23.5
Rather	12.1	8.9	35.9	28.7	17.8	15.0	13.8	8.5	19.8	17.0	21.8	25.5
Completely	1.7	2.8	19.8	18.2	4.0	7.7	2.7	2.8	6.0	4.9	13.1	9.3
OR t2 vs. t1	OR = 0.9, *p* = 0.5		OR = 0.8, *p* = 0.07		OR = 0.9, *p* = 0.7		OR = 0.7, *p* = 0.01		OR = 0.8, *p* = 0.07		OR = 0.8, *p* = 0.2	

	I am anxious (%)		I don't care (%)		Precautions are exaggerated (%)		I inform myself (%)		I miss my peers (%)		I'd rather go back to school (%)	
Answer	t1	t2	t1	t2	t1	t2	t1	t2	t1	t2	t1	t2
Not at all	30.5	36.4	11.4	8.5	50	47	16.1	17	6.7	6.9		8.5
A little	46.6	42.9	28.2	26.7	22.1	25.5	32.6	34	12.1	14.2		14.2
Moderately	17.8	16.6	38.3	37.2	15.4	16.2	17.1	19	20.1	19.8		19.8
Rather	3.7	3.2	16.8	19	9.1	10.1	24.8	19.4	27.9	32		25.5
Completely	1.3	0.8	5.4	8.5	3.4	1.2	9.4	10.5	33.2	27.1		32
OR t2 vs. t1	OR = 0.8, *p* = 0.2		OR = 1.3, *p* = 0.1		OR = 1.0, *p* = 0.7		OR = 0.9, *p* = 0.5		OR = 0.8, *p* = 0.3			

Abbreviation: OR, odds ratio.

23%/21% of the children stated that they were afraid of the Coronavirus at least moderately at t1/t2. About 50% informed themselves regularly about the current developments regarding the pandemic at t1/t2 (i.e., agreed at least moderately), and about 80% missed friends and peers. We could not find any differences between t1 and t2 for Question B (Table 3). Medium/low SES was significantly related to less interest in information about the pandemic (OR = 0.52, *p* < 0.001). Girls missed school more (OR = 1.96, *p* = 0.015) and were more afraid (OR = 1.55, *p* = 0.036) than boys. Older children were more interested in pandemic‐related information (OR = 1.18, *p* < 0.001), suffered less from the lack of real contacts (OR = 0.88, *p* < 0.001), and were less afraid (OR = 0.89, *p* = 0.002) than younger children.

The anticipated duration of the pandemic (Question C) increased significantly from t1 to t2 (OR = 1.76, *p* = 0.006). Whereas 7.4% of the children thought that it would never be as before the crisis at t1, the proportion raised to 16.2% (*p* < 0.001) at t2. The only predictor significantly related to the anticipated duration at t1 was age (OR = 1.14, *p* = 0.002).

### 3.3 Media use

**FIGURE 2:**
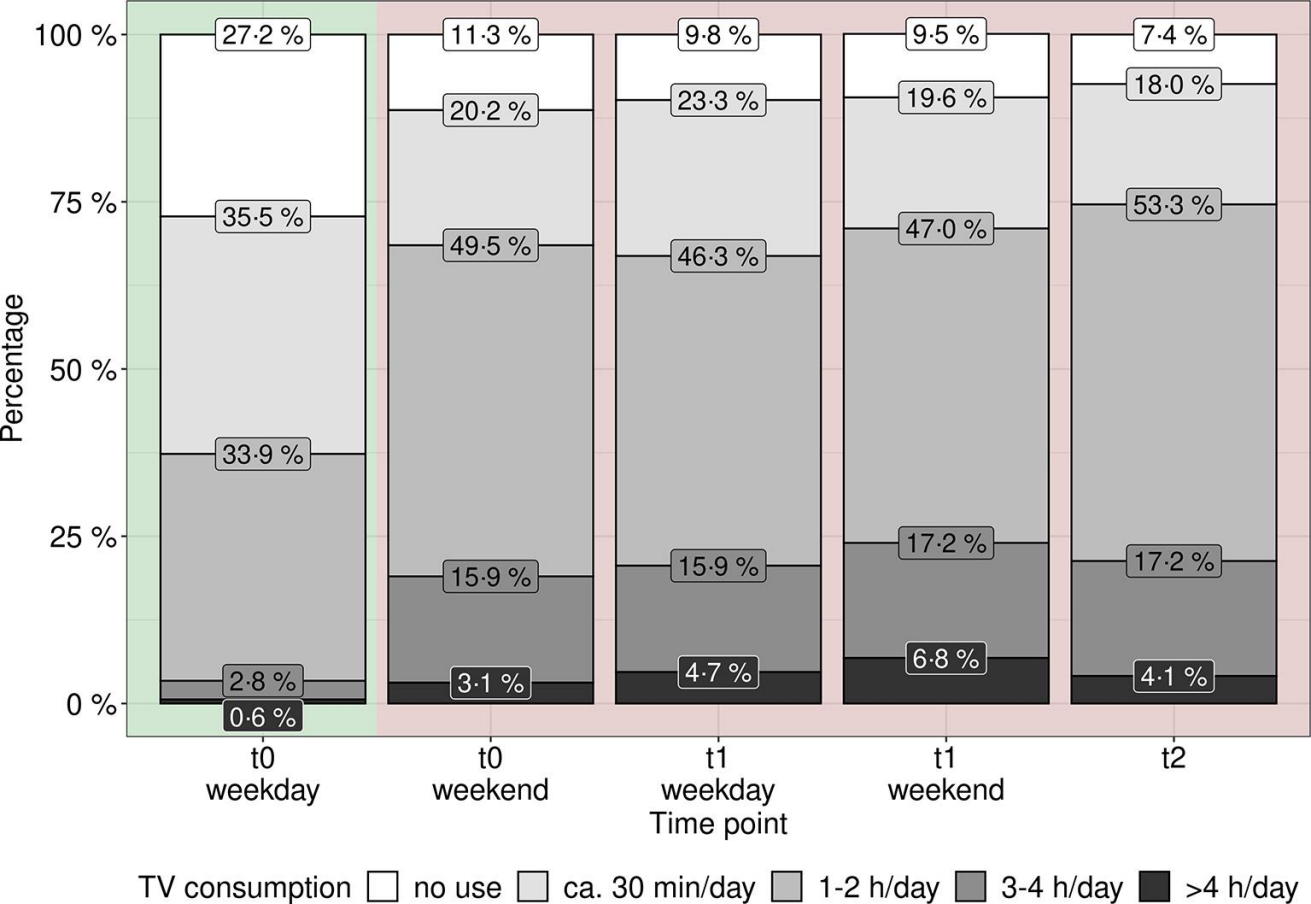
The TV/DVD/video use was significantly different between weekdays and weekends at t0 but not at t1. The TV/DVD/video consumption at t1 (weekdays as well as weekends) was similar to the use at weekends at t0. At t2, we only asked for TV/DVD/video use in general to minimize the proband's effort which is also similar to the consumption on weekends at t0

Before COVID‐19 (t0), the media use was significantly higher on weekends than on weekdays (Table S3). At t1, we could not find significant differences in media use between weekdays and weekends. Because the questions on media use differed between t0 and t1, we only could compare answers for TV/DVD/video consumption between t0 and t1. The OR for TV/DVD/video consumption on weekdays at t1 versus t0 was OR = 3.80 (*p* < 0.001), which is comparable to the OR weekend versus weekdays at t0 (OR = 3.77, *p* < 0.001). Therefore, the TV/DVD/video consumption at t1 (weekdays/weekends) is similar to the consumption at weekends at t0 (Figure 2).

## 4 DISCUSSION

We have found that the COVID‐19 pandemic and the measures against it affected the wellbeing and behavior of 9–18‐year‐old children. Psychological and physical well‐being and perceived social support decreased substantially shortly after the start of the lockdown compared to before COVID‐19. This is in line with Li et al. (2020), who reported an increase in negative emotions and a decrease in positive emotions among users of a social network during the evolving COVID‐19 epidemic in China.

Age was inversely correlated to the decrease in perceived social support, which might be linked to the higher availability and a more targeted use of electronic devices and social platforms with increasing age (Auhuber et al., 2019). Another reason might be noncompliance with social distancing guidelines in adolescents. Goldstein and Lipsitch (2020) reported a temporal rise in the proportion of infections in the 15–24‐year‐old population compared to the overall rate following the introduction of social distancing and discussed noncompliance as a likely reason. This hypothesis is supported by SteelFisher et al. (2012), who showed that social distancing was less accepted by adults than other protective measures during the 2009 H1N1 pandemic.

Most of the children stated that they only worry little or not at all about themselves. A reason might be the public discussion on children being less affected by COVID‐19. There is also evidence that, during an epidemic, people perceive themselves as less likely to be infected than other individuals (Bults et al., 2015). Contrastingly, most of the children and adolescents worried about their families, a fear that might be triggered by listening about the vulnerability of grandparents or other vulnerable family members and the resulting social distancing (Weaver & Wiener, 2020).

We found girls experience more anxiety than boys which is in line with McElroy et al. (2020) and Zhou et al. (2020). Consistently they worried more about themselves, friends and family, their town, the country and the world although not all of the effects reached statistical significance. However, the highest effects were found for worries with a societal scope. The finding that girls have greater concerns about environmental (in contrast so personal) concerns is in line with Henker et al. (1995). Moreover, Anttila et al. (2000) reported that worries about global threats were the most important group of worries in 825 16–18 Finnish high school girls.

The observed increase in media use supports the results of Xiang et al. (2020) and Pietrobelli et al. (2020). Both reported a substantial increase in screen time during the COVID‐19 pandemic. The increased media use might partly be induced by the increased dependence on electronic media when it comes to school work and social contacts but also by a lack of daily structure. The latter is known to foster unhealthy behaviors like unhealthy eating habits and an increase in sedentary activities (Brazendale et al., 2017; Hippel et al., 2007). Increased media use is only one aspect of behavioral change. Several studies showed that the increase in screen time was paralleled by a decrease in PA (Dunton et al., 2020; Moore et al., 2020; Pietrobelli et al., 2020; Xiang et al., 2020; Zheng, 2020), which is exacerbated by the closing of sports clubs and fitness facilities. Unfortunately, we have no data about this aspect of the children's lives.

### 4.1 Limitations

The underrepresentation of low SES might cause an underestimation of effects because, as several studies show, calamities' adverse effects are more significant in vulnerable groups (Loades et al., 2020). Another limitation is the response rate of about 50%–60%. Besides, our analyses did not include PA measures, and the questions on media use were not identical at t0 and t1/t2. Furthermore, the findings may not generalize to other countries and/or other areas of Germany since the area around Leipzig was only mildly affected by the crises.

Even though the study region was only mildly affected by the virus, public life was shut down, and children were not allowed to meet with their friends or visit their grandparents. We have found that the measures had a negative impact on the well‐being and health‐related behavior of children and tended to be more aggravated in socioeconomically disadvantaged families. Our results support the notion that measures against a pandemic have to be balanced against adverse public health effects, especially vulnerable groups like children and adolescents have to be protected as they are more susceptible to the negative consequences.

6

## 7 AUTHOR CONTRIBUTION STATEMENT

MV and TP devised the project and the main conceptual ideas. MV drafted the work and analyzed and interpreted the data and designed the figures; MV, WK, and TP discussed and finalized the first draft of the manuscript; MV, TP, CS, PO, UI, CM, AK, and WK revised the work critically for important intellectual content and discussed the results; WK, CM, MV, CS, PO, and TP contributed to the conception and design of data collection; CM designed and maintained the software system used for data collection and data management; MV and CM are responsible for data collection and data quality; all authors approved the final version of the manuscript. AK and WK are the principal investigators of the LIFE Child study.

## Supporting information

SUPPORTING INFORMATION 1

## Data Availability

The LIFE Child study is a study collecting potentially sensitive information. Data cannot be shared publicly because there exist ethical and legal restrictions. Publishing data sets is not covered by the informed consent provided by the study participants. However, every researcher affiliated with a research institution can request data access. Researchers interested in accessing and analyzing data collected in the LIFE Child study may contact the data use and access committee (dm@life.uni-leipzig.de).
