# Supplementary material for: Well‐being and COVID‐19‐related worries of German children and adolescents: A longitudinal study from pre‐COVID to the end of lockdown in Spring 2020
Source: JCPP Adv. 2021 Mar 17;1(1):e12004. doi: 10.1111/jcv2.12004 (PMC8250267; doi:10.1111/jcv2.12004)
Supplement: Supplementary file 1 — SUPPORTING INFORMATION 1 [file JCV2-1-e12004-s001.docx]

**Table S1:** Comparison of variables regarding the participation only at t1, only at t2, or at both t1 and t2. Raw p-values and the p-values adjusted for multiple testing are reported.

|  | Only t1 N=132 | T1 and t2 N=189 | Only t2 N=70 | p raw | p ajusted |
| --- | --- | --- | --- | --- | --- |
| Age t0 (years) | 12.6 (2.43) | 12.5 (2.53) | 12.2 (2.29) | 0.483 | 0.797 |
| Age t1 (years) | 13.2 (2.43) | 13.3 (2.51) |  | 0.816 | 0.938 |
| Age t2 (years) |  | 13.3 (2.51) | 12.9 (2.31) | 0.237 | 0.735 |
| Participants at t1: |  |  |  | <0.001 | <0.001 |
| yes | 130 (100%) | 187 (100%) | 0 (0.00%) |  |  |
| no | 0 (0.00%) | 0 (0.00%) | 70 (100%) |  |  |
| Participants at t2: |  |  |  | <0.001 | <0.001 |
| yes | 0 (0.00%) | 187 (100%) | 70 (100%) |  |  |
| no | 130 (100%) | 0 (0.00%) | 0 (0.00%) |  |  |
| Time difference t0->t1 (years) | 0.75 (0.31) | 0.75 (0.32) | 0.75 (0.32) | 0.979 | 0.979 |
| Socioeconomic status: |  |  |  | 0.329 | 0.797 |
| low | 3 (2.31%) | 2 (1.07%) | 0 (0.00%) |  |  |
| middle | 63 (48.5%) | 95 (50.8%) | 39 (55.7%) |  |  |
| high | 58 (44.6%) | 86 (46.0%) | 26 (37.1%) |  |  |
| missing | 6 (4.62%) | 4 (2.14%) | 5 (7.14%) |  |  |
| Physical wellbeing at t0 | 47.5 (6.78) | 50.0 (5.72) | 49.6 (6.08) | 0.002 | 0.051 |
| Physical wellbeing at t1 | 46.1 (7.40) | 45.4 (6.20) |  | 0.418 | 0.797 |
| Physical wellbeing at t2 |  | 46.0 (6.54) | 46.2 (7.57) | 0.789 | 0.933 |
| Psychological wellbeing at t0 | 38.9 (6.08) | 38.2 (3.64) | 37.9 (2.65) | 0.312 | 0.797 |
| Psychological wellbeing at t1 | 36.7 (2.37) | 36.2 (2.56) |  | 0.091 | 0.493 |
| Psychological wellbeing at t2 |  | 36.4 (2.74) | 36.2 (2.29) | 0.584 | 0.863 |
| Peers & social support at t0 | 52.6 (11.3) | 52.2 (10.4) | 54.3 (10.7) | 0.403 | 0.797 |
| Peers & social support at t1 | 46.0 (10.2) | 47.0 (8.90) |  | 0.399 | 0.797 |
| Peers & social support at t2 |  | 47.1 (8.40) | 48.9 (9.91) | 0.185 | 0.631 |
| TV/DVD/Streaming weekdays at t1: |  |  |  | 0.543 | 0.829 |
| Mehr als 4 Stunden pro Tag | 6 (4.96%) | 8 (4.57%) |  |  |  |
| ungefähr 3-4 Stunden pro Tag | 20 (16.5%) | 27 (15.4%) |  |  |  |
| ungefähr 1-2 Stunden pro Tag | 55 (45.5%) | 82 (46.9%) |  |  |  |
| ungefähr 30 Minuten pro Tag | 32 (26.4%) | 37 (21.1%) |  |  |  |
| gar nicht | 8 (6.61%) | 21 (12.0%) |  |  |  |
| TV/DVD/Streaming weekends at t1: |  |  |  | 0.504 | 0.799 |
| Mehr als 4 Stunden pro Tag | 10 (8.26%) | 10 (5.71%) |  |  |  |
| ungefähr 3-4 Stunden pro Tag | 17 (14.0%) | 34 (19.4%) |  |  |  |
| ungefähr 1-2 Stunden pro Tag | 55 (45.5%) | 84 (48.0%) |  |  |  |
| ungefähr 30 Minuten pro Tag | 28 (23.1%) | 30 (17.1%) |  |  |  |
| gar nicht | 11 (9.09%) | 17 (9.71%) |  |  |  |
| TV/DVD/Streaming at t2: |  |  |  | 0.746 | 0.922 |
| Mehr als 4 Stunden pro Tag | 0 (.%) | 7 (3.89%) | 3 (4.69%) |  |  |
| ungefähr 3-4 Stunden pro Tag | 0 (.%) | 33 (18.3%) | 9 (14.1%) |  |  |
| ungefähr 1-2 Stunden pro Tag | 0 (.%) | 92 (51.1%) | 38 (59.4%) |  |  |
| ungefähr 30 Minuten pro Tag | 0 (.%) | 35 (19.4%) | 9 (14.1%) |  |  |
| gar nicht | 0 (.%) | 13 (7.22%) | 5 (7.81%) |  |  |
| Making Videos weekdays at t1: |  |  |  | 0.167 | 0.624 |
| ungefähr 1-2 Stunden pro Tag | 7 (5.79%) | 10 (5.71%) |  |  |  |
| ungefähr 30 Minuten pro Tag | 23 (19.0%) | 50 (28.6%) |  |  |  |
| gar nicht | 91 (75.2%) | 115 (65.7%) |  |  |  |
| Making Videos weekends at t1: |  |  |  | 0.452 | 0.797 |
| ungefähr 1-2 Stunden pro Tag | 7 (5.79%) | 11 (6.29%) |  |  |  |
| ungefähr 30 Minuten pro Tag | 27 (22.3%) | 50 (28.6%) |  |  |  |
| gar nicht | 87 (71.9%) | 114 (65.1%) |  |  |  |
| Making Videos at t2: |  |  |  | 0.667 | 0.904 |
| ungefähr 3-4 Stunden pro Tag |  | 1 (0.56%) | 0 (0.00%) |  |  |
| ungefähr 1-2 Stunden pro Tag |  | 10 (5.56%) | 6 (9.38%) |  |  |
| ungefähr 30 Minuten pro Tag |  | 45 (25.0%) | 14 (21.9%) |  |  |
| gar nicht |  | 124 (68.9%) | 44 (68.8%) |  |  |
| Computer Games weekdays at t1: |  |  |  | 0.659 | 0.904 |
| Mehr als 4 Stunden pro Tag | 13 (10.7%) | 16 (9.14%) |  |  |  |
| ungefähr 3-4 Stunden pro Tag | 17 (14.0%) | 21 (12.0%) |  |  |  |
| ungefähr 1-2 Stunden pro Tag | 39 (32.2%) | 48 (27.4%) |  |  |  |
| ungefähr 30 Minuten pro Tag | 29 (24.0%) | 55 (31.4%) |  |  |  |
| gar nicht | 23 (19.0%) | 35 (20.0%) |  |  |  |
| Computer Games weekends at t1: |  |  |  | 0.822 | 0.938 |
| Mehr als 4 Stunden pro Tag | 13 (10.7%) | 17 (9.71%) |  |  |  |
| ungefähr 3-4 Stunden pro Tag | 23 (19.0%) | 26 (14.9%) |  |  |  |
| ungefähr 1-2 Stunden pro Tag | 38 (31.4%) | 53 (30.3%) |  |  |  |
| ungefähr 30 Minuten pro Tag | 24 (19.8%) | 41 (23.4%) |  |  |  |
| gar nicht | 23 (19.0%) | 38 (21.7%) |  |  |  |
| Computer Games at t2: |  |  |  | 0.858 | 0.961 |
| Mehr als 4 Stunden pro Tag |  | 12 (6.67%) | 5 (7.81%) |  |  |
| ungefähr 3-4 Stunden pro Tag |  | 22 (12.2%) | 8 (12.5%) |  |  |
| ungefähr 1-2 Stunden pro Tag |  | 64 (35.6%) | 25 (39.1%) |  |  |
| ungefähr 30 Minuten pro Tag |  | 46 (25.6%) | 12 (18.8%) |  |  |
| gar nicht |  | 36 (20.0%) | 14 (21.9%) |  |  |
| Social Media (active) weekdays at t1: |  |  |  | 0.975 | 0.979 |
| Mehr als 4 Stunden pro Tag | 5 (4.13%) | 7 (4.00%) |  |  |  |
| ungefähr 3-4 Stunden pro Tag | 13 (10.7%) | 17 (9.71%) |  |  |  |
| ungefähr 1-2 Stunden pro Tag | 38 (31.4%) | 57 (32.6%) |  |  |  |
| ungefähr 30 Minuten pro Tag | 37 (30.6%) | 58 (33.1%) |  |  |  |
| gar nicht | 28 (23.1%) | 36 (20.6%) |  |  |  |
| Social Media (active) weekends at t1: |  |  |  | 0.949 | 0.979 |
| Mehr als 4 Stunden pro Tag | 7 (5.79%) | 8 (4.57%) |  |  |  |
| ungefähr 3-4 Stunden pro Tag | 10 (8.26%) | 16 (9.14%) |  |  |  |
| ungefähr 1-2 Stunden pro Tag | 41 (33.9%) | 56 (32.0%) |  |  |  |
| ungefähr 30 Minuten pro Tag | 35 (28.9%) | 57 (32.6%) |  |  |  |
| gar nicht | 28 (23.1%) | 38 (21.7%) |  |  |  |
| Social Media (active) at t2: |  |  |  | 0.491 | 0.797 |
| Mehr als 4 Stunden pro Tag |  | 8 (4.44%) | 3 (4.69%) |  |  |
| ungefähr 3-4 Stunden pro Tag |  | 6 (3.33%) | 4 (6.25%) |  |  |
| ungefähr 1-2 Stunden pro Tag |  | 54 (30.0%) | 14 (21.9%) |  |  |
| ungefähr 30 Minuten pro Tag |  | 74 (41.1%) | 25 (39.1%) |  |  |
| gar nicht |  | 38 (21.1%) | 18 (28.1%) |  |  |
| Social Media (passive) weekdays at t1: |  |  |  | 0.145 | 0.624 |
| Mehr als 4 Stunden pro Tag | 6 (4.96%) | 4 (2.29%) |  |  |  |
| ungefähr 3-4 Stunden pro Tag | 11 (9.09%) | 27 (15.4%) |  |  |  |
| ungefähr 1-2 Stunden pro Tag | 44 (36.4%) | 72 (41.1%) |  |  |  |
| ungefähr 30 Minuten pro Tag | 42 (34.7%) | 43 (24.6%) |  |  |  |
| gar nicht | 18 (14.9%) | 29 (16.6%) |  |  |  |
| Social Media (passive) weekends at t1: |  |  |  | 0.034 | 0.318 |
| Mehr als 4 Stunden pro Tag | 7 (5.79%) | 8 (4.57%) |  |  |  |
| ungefähr 3-4 Stunden pro Tag | 15 (12.4%) | 29 (16.6%) |  |  |  |
| ungefähr 1-2 Stunden pro Tag | 41 (33.9%) | 72 (41.1%) |  |  |  |
| ungefähr 30 Minuten pro Tag | 45 (37.2%) | 37 (21.1%) |  |  |  |
| gar nicht | 13 (10.7%) | 29 (16.6%) |  |  |  |
| Social Media (passive) at t2: |  |  |  | 0.380 | 0.797 |
| Mehr als 4 Stunden pro Tag |  | 11 (6.11%) | 2 (3.12%) |  |  |
| ungefähr 3-4 Stunden pro Tag |  | 24 (13.3%) | 4 (6.25%) |  |  |
| ungefähr 1-2 Stunden pro Tag |  | 80 (44.4%) | 30 (46.9%) |  |  |
| ungefähr 30 Minuten pro Tag |  | 34 (18.9%) | 12 (18.8%) |  |  |
| gar nicht |  | 31 (17.2%) | 16 (25.0%) |  |  |
| Reading Books weekdays at t1: |  |  |  | 0.434 | 0.797 |
| Mehr als 4 Stunden pro Tag | 1 (0.83%) | 3 (1.71%) |  |  |  |
| ungefähr 3-4 Stunden pro Tag | 5 (4.13%) | 3 (1.71%) |  |  |  |
| ungefähr 1-2 Stunden pro Tag | 23 (19.0%) | 44 (25.1%) |  |  |  |
| ungefähr 30 Minuten pro Tag | 53 (43.8%) | 78 (44.6%) |  |  |  |
| gar nicht | 39 (32.2%) | 47 (26.9%) |  |  |  |
| Reading Books weekends at t1: |  |  |  | 0.702 | 0.922 |
| Mehr als 4 Stunden pro Tag | 1 (0.83%) | 4 (2.29%) |  |  |  |
| ungefähr 3-4 Stunden pro Tag | 5 (4.13%) | 8 (4.57%) |  |  |  |
| ungefähr 1-2 Stunden pro Tag | 24 (19.8%) | 43 (24.6%) |  |  |  |
| ungefähr 30 Minuten pro Tag | 53 (43.8%) | 74 (42.3%) |  |  |  |
| gar nicht | 38 (31.4%) | 46 (26.3%) |  |  |  |
| Reading Books at t2: |  |  |  | 0.382 | 0.797 |
| Mehr als 4 Stunden pro Tag | 0 (.%) | 3 (1.67%) | 2 (3.12%) |  |  |
| ungefähr 3-4 Stunden pro Tag | 0 (.%) | 8 (4.44%) | 0 (0.00%) |  |  |
| ungefähr 1-2 Stunden pro Tag | 0 (.%) | 49 (27.2%) | 18 (28.1%) |  |  |
| ungefähr 30 Minuten pro Tag | 0 (.%) | 68 (37.8%) | 28 (43.8%) |  |  |
| gar nicht | 0 (.%) | 52 (28.9%) | 16 (25.0%) |  |  |
| Reading Magazins weekdays at t1: |  |  |  | 0.948 | 0.979 |
| ungefähr 3-4 Stunden pro Tag | 0 (0.00%) | 1 (0.57%) |  |  |  |
| ungefähr 1-2 Stunden pro Tag | 7 (5.79%) | 8 (4.57%) |  |  |  |
| ungefähr 30 Minuten pro Tag | 31 (25.6%) | 46 (26.3%) |  |  |  |
| gar nicht | 83 (68.6%) | 120 (68.6%) |  |  |  |
| Reading Magazins weekends at t1: |  |  |  | 0.779 | 0.933 |
| ungefähr 3-4 Stunden pro Tag | 0 (0.00%) | 1 (0.57%) |  |  |  |
| ungefähr 1-2 Stunden pro Tag | 8 (6.61%) | 8 (4.57%) |  |  |  |
| ungefähr 30 Minuten pro Tag | 33 (27.3%) | 44 (25.1%) |  |  |  |
| gar nicht | 80 (66.1%) | 122 (69.7%) |  |  |  |
| Reading Magazins at t2: |  |  |  | 0.655 | 0.904 |
| Mehr als 4 Stunden pro Tag | 0 (.%) | 1 (0.56%) | 0 (0.00%) |  |  |
| ungefähr 1-2 Stunden pro Tag | 0 (.%) | 10 (5.56%) | 5 (7.81%) |  |  |
| ungefähr 30 Minuten pro Tag | 0 (.%) | 51 (28.3%) | 14 (21.9%) |  |  |
| gar nicht | 0 (.%) | 118 (65.6%) | 45 (70.3%) |  |  |
| Reading with Parents weekdays at t1: |  |  |  | 0.048 | 0.390 |
| ungefähr 1-2 Stunden pro Tag | 0 (0.00%) | 8 (4.57%) |  |  |  |
| ungefähr 30 Minuten pro Tag | 25 (20.7%) | 33 (18.9%) |  |  |  |
| gar nicht | 96 (79.3%) | 134 (76.6%) |  |  |  |
| Reading with Parents weekends at t1: |  |  |  | 0.060 | 0.979 |
| ungefähr 1-2 Stunden pro Tag | 1 (0.83%) | 11 (6.29%) |  |  |  |
| ungefähr 30 Minuten pro Tag | 26 (21.5%) | 35 (20.0%) |  |  |  |
| gar nicht | 94 (77.7%) | 129 (73.7%) |  |  |  |
| Reading with Parents at t2: |  |  |  | 0.912 | 0.797 |
| ungefähr 1-2 Stunden pro Tag |  | 6 (3.33%) | 1 (1.56%) |  |  |
| ungefähr 30 Minuten pro Tag |  | 39 (21.7%) | 14 (21.9%) |  |  |
| gar nicht |  | 135 (75.0%) | 49 (76.6%) |  |  |
| Music Radio weekdays at t1: |  |  |  | 0.291 | 0.797 |
| Mehr als 4 Stunden pro Tag | 13 (10.7%) | 16 (9.14%) |  |  |  |
| ungefähr 3-4 Stunden pro Tag | 16 (13.2%) | 17 (9.71%) |  |  |  |
| ungefähr 1-2 Stunden pro Tag | 49 (40.5%) | 71 (40.6%) |  |  |  |
| ungefähr 30 Minuten pro Tag | 37 (30.6%) | 50 (28.6%) |  |  |  |
| gar nicht | 6 (4.96%) | 21 (12.0%) |  |  |  |
| Music Radio weekends at t1: |  |  |  | 0.011 | 0.180 |
| Mehr als 4 Stunden pro Tag | 18 (14.9%) | 14 (8.00%) |  |  |  |
| ungefähr 3-4 Stunden pro Tag | 14 (11.6%) | 21 (12.0%) |  |  |  |
| ungefähr 1-2 Stunden pro Tag | 48 (39.7%) | 68 (38.9%) |  |  |  |
| ungefähr 30 Minuten pro Tag | 37 (30.6%) | 46 (26.3%) |  |  |  |
| gar nicht | 4 (3.31%) | 26 (14.9%) |  |  |  |
| Music Radio at t2: |  |  |  | 0.173 | 0.624 |
| Mehr als 4 Stunden pro Tag |  | 17 (9.44%) | 7 (10.9%) |  |  |
| ungefähr 3-4 Stunden pro Tag |  | 21 (11.7%) | 13 (20.3%) |  |  |
| ungefähr 1-2 Stunden pro Tag |  | 66 (36.7%) | 27 (42.2%) |  |  |
| ungefähr 30 Minuten pro Tag |  | 57 (31.7%) | 14 (21.9%) |  |  |
| gar nicht |  | 19 (10.6%) | 3 (4.69%) |  |  |
| Jigsaw/Drawing at t1: |  |  |  | 0.467 | 0.797 |
| mindestens 1x pro Tag | 26 (21.5%) | 30 (17.1%) |  |  |  |
| mindestens 3x pro Woche | 9 (7.44%) | 23 (13.1%) |  |  |  |
| mindestens 1x pro Woche | 25 (20.7%) | 32 (18.3%) |  |  |  |
| seltener als 1x pro Woche | 27 (22.3%) | 45 (25.7%) |  |  |  |
| nie | 34 (28.1%) | 45 (25.7%) |  |  |  |
| Jigsaw/Drawing at t2: |  |  |  | 0.548 | 0.829 |
| mindestens 1x pro Tag |  | 21 (11.6%) | 10 (15.4%) |  |  |
| mindestens 3x pro Woche |  | 29 (16.0%) | 11 (16.9%) |  |  |
| mindestens 1x pro Woche |  | 43 (23.8%) | 20 (30.8%) |  |  |
| seltener als 1x pro Woche |  | 40 (22.1%) | 12 (18.5%) |  |  |
| nie |  | 48 (26.5%) | 12 (18.5%) |  |  |
| Parlor Games at t1: |  |  |  | 0.466 | 0.797 |
| mindestens 1x pro Tag | 8 (6.61%) | 17 (9.71%) |  |  |  |
| mindestens 3x pro Woche | 17 (14.0%) | 35 (20.0%) |  |  |  |
| mindestens 1x pro Woche | 38 (31.4%) | 53 (30.3%) |  |  |  |
| seltener als 1x pro Woche | 34 (28.1%) | 38 (21.7%) |  |  |  |
| nie | 24 (19.8%) | 32 (18.3%) |  |  |  |
| Parlor Games at t2: |  |  |  | 0.612 | 0.884 |
| mindestens 1x pro Tag |  | 12 (6.63%) | 5 (7.69%) |  |  |
| mindestens 3x pro Woche |  | 24 (13.3%) | 12 (18.5%) |  |  |
| mindestens 1x pro Woche |  | 59 (32.6%) | 15 (23.1%) |  |  |
| seltener als 1x pro Woche |  | 46 (25.4%) | 18 (27.7%) |  |  |
| nie |  | 40 (22.1%) | 15 (23.1%) |  |  |
| Playing with Pets at t1: |  |  |  | 0.217 | 0.705 |
| mindestens 1x pro Tag | 30 (24.8%) | 49 (28.0%) |  |  |  |
| mindestens 3x pro Woche | 16 (13.2%) | 16 (9.14%) |  |  |  |
| mindestens 1x pro Woche | 13 (10.7%) | 15 (8.57%) |  |  |  |
| seltener als 1x pro Woche | 9 (7.44%) | 5 (2.86%) |  |  |  |
| nie | 53 (43.8%) | 90 (51.4%) |  |  |  |
| Playing with Pets at t2: |  |  |  | 0.730 | 0.922 |
| mindestens 1x pro Tag |  | 43 (23.8%) | 20 (30.8%) |  |  |
| mindestens 3x pro Woche |  | 32 (17.7%) | 11 (16.9%) |  |  |
| mindestens 1x pro Woche |  | 19 (10.5%) | 4 (6.15%) |  |  |
| seltener als 1x pro Woche |  | 7 (3.87%) | 3 (4.62%) |  |  |
| nie |  | 80 (44.2%) | 27 (41.5%) |  |  |
| Cleaning at t1: |  |  |  | 0.952 | 0.979 |
| mindestens 1x pro Tag | 10 (8.26%) | 13 (7.43%) |  |  |  |
| mindestens 3x pro Woche | 23 (19.0%) | 32 (18.3%) |  |  |  |
| mindestens 1x pro Woche | 45 (37.2%) | 72 (41.1%) |  |  |  |
| seltener als 1x pro Woche | 30 (24.8%) | 38 (21.7%) |  |  |  |
| nie | 13 (10.7%) | 20 (11.4%) |  |  |  |
| Cleaning at t2: |  |  |  | 0.307 | 0.797 |
| mindestens 1x pro Tag |  | 19 (10.5%) | 7 (10.8%) |  |  |
| mindestens 3x pro Woche |  | 18 (9.94%) | 11 (16.9%) |  |  |
| mindestens 1x pro Woche |  | 82 (45.3%) | 21 (32.3%) |  |  |
| seltener als 1x pro Woche |  | 46 (25.4%) | 21 (32.3%) |  |  |
| nie |  | 16 (8.84%) | 5 (7.69%) |  |  |
| Technical Toys at t1: |  |  |  | 0.350 | 0.797 |
| mindestens 1x pro Tag | 16 (13.2%) | 13 (7.43%) |  |  |  |
| mindestens 3x pro Woche | 10 (8.26%) | 10 (5.71%) |  |  |  |
| mindestens 1x pro Woche | 12 (9.92%) | 18 (10.3%) |  |  |  |
| seltener als 1x pro Woche | 17 (14.0%) | 34 (19.4%) |  |  |  |
| nie | 66 (54.5%) | 100 (57.1%) |  |  |  |
| Technical Toys at t2: |  |  |  | 0.420 | 0.797 |
| mindestens 1x pro Tag |  | 9 (4.97%) | 8 (12.3%) |  |  |
| mindestens 3x pro Woche |  | 14 (7.73%) | 5 (7.69%) |  |  |
| mindestens 1x pro Woche |  | 26 (14.4%) | 9 (13.8%) |  |  |
| seltener als 1x pro Woche |  | 30 (16.6%) | 10 (15.4%) |  |  |
| nie |  | 102 (56.4%) | 33 (50.8%) |  |  |
| Indoor Sports at t1: |  |  |  | 0.904 | 0.979 |
| mindestens 1x pro Tag | 30 (24.8%) | 45 (25.7%) | 0 (.%) |  |  |
| mindestens 3x pro Woche | 24 (19.8%) | 37 (21.1%) | 0 (.%) |  |  |
| mindestens 1x pro Woche | 24 (19.8%) | 40 (22.9%) | 0 (.%) |  |  |
| seltener als 1x pro Woche | 22 (18.2%) | 26 (14.9%) | 0 (.%) |  |  |
| nie | 21 (17.4%) | 27 (15.4%) | 0 (.%) |  |  |
| Indoor Sports at t2: |  |  |  | 0.079 | 0.470 |
| mindestens 1x pro Tag |  | 29 (16.0%) | 21 (32.3%) |  |  |
| mindestens 3x pro Woche |  | 44 (24.3%) | 11 (16.9%) |  |  |
| mindestens 1x pro Woche |  | 52 (28.7%) | 16 (24.6%) |  |  |
| seltener als 1x pro Woche |  | 24 (13.3%) | 6 (9.23%) |  |  |
| nie |  | 32 (17.7%) | 11 (16.9%) |  |  |
| Outdoor Sports at t1: |  |  |  | 0.024 | 0.316 |
| mindestens 1x pro Tag | 30 (24.8%) | 26 (14.9%) |  |  |  |
| mindestens 3x pro Woche | 20 (16.5%) | 55 (31.4%) |  |  |  |
| mindestens 1x pro Woche | 30 (24.8%) | 39 (22.3%) |  |  |  |
| seltener als 1x pro Woche | 18 (14.9%) | 19 (10.9%) |  |  |  |
| nie | 23 (19.0%) | 36 (20.6%) |  |  |  |
| Outdoor Sports at t2: |  |  |  | 0.053 | 0.382 |
| mindestens 1x pro Tag |  | 34 (18.8%) | 15 (23.1%) |  |  |
| mindestens 3x pro Woche |  | 44 (24.3%) | 21 (32.3%) |  |  |
| mindestens 1x pro Woche |  | 39 (21.5%) | 19 (29.2%) |  |  |
| seltener als 1x pro Woche |  | 32 (17.7%) | 4 (6.15%) |  |  |
| nie |  | 32 (17.7%) | 6 (9.23%) |  |  |
| Walking/Biking at t1: |  |  |  | 0.160 | 0.624 |
| mindestens 1x pro Tag | 30 (24.8%) | 39 (22.3%) |  |  |  |
| mindestens 3x pro Woche | 29 (24.0%) | 50 (28.6%) |  |  |  |
| mindestens 1x pro Woche | 25 (20.7%) | 50 (28.6%) |  |  |  |
| seltener als 1x pro Woche | 21 (17.4%) | 16 (9.14%) |  |  |  |
| nie | 16 (13.2%) | 20 (11.4%) |  |  |  |
| Walking/Biking at t2: |  |  |  | 0.034 | 0.318 |
| mindestens 1x pro Tag |  | 38 (21.0%) | 27 (41.5%) |  |  |
| mindestens 3x pro Woche |  | 64 (35.4%) | 19 (29.2%) |  |  |
| mindestens 1x pro Woche |  | 45 (24.9%) | 11 (16.9%) |  |  |
| seltener als 1x pro Woche |  | 25 (13.8%) | 5 (7.69%) |  |  |
| nie |  | 9 (4.97%) | 3 (4.62%) |  |  |
| Cooking/Baking at t1: |  |  |  | 0.745 | 0.922 |
| mindestens 1x pro Tag | 9 (7.44%) | 19 (10.9%) |  |  |  |
| mindestens 3x pro Woche | 30 (24.8%) | 37 (21.1%) |  |  |  |
| mindestens 1x pro Woche | 36 (29.8%) | 46 (26.3%) |  |  |  |
| seltener als 1x pro Woche | 29 (24.0%) | 44 (25.1%) |  |  |  |
| nie | 17 (14.0%) | 29 (16.6%) |  |  |  |
| Cooking/Baking at t2: |  |  |  | 0.439 | 0.797 |
| mindestens 1x pro Tag |  | 16 (8.84%) | 6 (9.23%) |  |  |
| mindestens 3x pro Woche |  | 31 (17.1%) | 14 (21.5%) |  |  |
| mindestens 1x pro Woche |  | 53 (29.3%) | 15 (23.1%) |  |  |
| seltener als 1x pro Woche |  | 46 (25.4%) | 22 (33.8%) |  |  |
| nie |  | 35 (19.3%) | 8 (12.3%) |  |  |
| Playing an Instrument at t1: |  |  |  | 0.414 | 0.797 |
| mindestens 1x pro Tag | 8 (6.61%) | 22 (12.6%) |  |  |  |
| mindestens 3x pro Woche | 15 (12.4%) | 27 (15.4%) |  |  |  |
| mindestens 1x pro Woche | 12 (9.92%) | 18 (10.3%) |  |  |  |
| seltener als 1x pro Woche | 10 (8.26%) | 13 (7.43%) |  |  |  |
| nie | 76 (62.8%) | 95 (54.3%) |  |  |  |
| Playing an Instrument at t2: |  |  |  | 0.172 | 0.624 |
| mindestens 1x pro Tag |  | 19 (10.5%) | 4 (6.15%) |  |  |
| mindestens 3x pro Woche |  | 27 (14.9%) | 11 (16.9%) |  |  |
| mindestens 1x pro Woche |  | 18 (9.94%) | 10 (15.4%) |  |  |
| seltener als 1x pro Woche |  | 16 (8.84%) | 1 (1.54%) |  |  |
| nie |  | 101 (55.8%) | 39 (60.0%) |  |  |
| Playing Outside at t1: |  |  |  | 0.157 | 0.624 |
| mindestens 1x pro Tag | 33 (27.3%) | 45 (25.7%) |  |  |  |
| mindestens 3x pro Woche | 12 (9.92%) | 36 (20.6%) |  |  |  |
| mindestens 1x pro Woche | 14 (11.6%) | 21 (12.0%) |  |  |  |
| seltener als 1x pro Woche | 17 (14.0%) | 22 (12.6%) |  |  |  |
| nie | 45 (37.2%) | 51 (29.1%) |  |  |  |
| Playing Outside at t2: |  |  |  | 0.752 | 0.922 |
| mindestens 1x pro Tag |  | 57 (31.5%) | 24 (36.9%) |  |  |
| mindestens 3x pro Woche |  | 37 (20.4%) | 10 (15.4%) |  |  |
| mindestens 1x pro Woche |  | 22 (12.2%) | 10 (15.4%) |  |  |
| seltener als 1x pro Woche |  | 26 (14.4%) | 7 (10.8%) |  |  |
| nie |  | 39 (21.5%) | 14 (21.5%) |  |  |

**Table S2:** Comparison of the three KIDSCREEN scales: effects, lower and higher confidence limits (cl) and p-values for the paired and unpaired tests. The scores of the three scales were significantly lower at t1 and t2 (during the COVID-19 lockdown) compared to t0 (before COVID-19). There was no difference between t1 and t2.

| scale | comparison | estimate | confidence interval | p.value | method |
| --- | --- | --- | --- | --- | --- |
| Peers/social support | t1 vs. t0 | -10,66 | [-12.5,-8.8] | p<0.001 | Paired t-test |
|  | t1 vs. t0 | -11,00 | [-13.0,-9.0] | p<0.001 | Welch Two Sample t-test |
|  | t2 vs. t0 | -9,70 | [-11.6,-7.8] | p<0.001 | Paired t-test |
|  | t2 vs. t0 | -9,89 | [-12.0,-7.7] | p<0.001 | Welch Two Sample t-test |
|  | t2 vs. t1 | 0,92 | [-1.2,3.0] | p=0.385 | Paired t-test |
|  | t2 vs. t1 | 1,11 | [-1.4,3.6] | p=0.387 | Welch Two Sample t-test |
| Physical wellbing | t1 vs. t0 | -3,30 | [-4.2,-2.4] | p<0.001 | Paired t-test |
|  | t1 vs. t0 | -3,40 | [-4.4,-2.4] | p<0.001 | Welch Two Sample t-test |
|  | t2 vs. t0 | -3,64 | [-4.6,-2.7] | p<0.001 | Paired t-test |
|  | t2 vs. t0 | -3,03 | [-4.1,-2.0] | p<0.001 | Welch Two Sample t-test |
|  | t2 vs. t1 | 0,51 | [-0.4,1.4] | p=0.247 | Paired t-test |
|  | t2 vs. t1 | 0,37 | [-0.8,1.5] | p=0.531 | Welch Two Sample t-test |
| Psychological wellbing | t1 vs. t0 | -2,04 | [-2.7,-1.4] | p<0.001 | Paired t-test |
|  | t1 vs. t0 | -1,95 | [-2.5,-1.4] | p<0.001 | Welch Two Sample t-test |
|  | t2 vs. t0 | -1,84 | [-2.3,-1.4] | p<0.001 | Paired t-test |
|  | t2 vs. t0 | -2,05 | [-2.6,-1.5] | p<0.001 | Welch Two Sample t-test |
|  | t2 vs. t1 | 0,10 | [-0.4,0.6] | p=0.703 | Paired t-test |
|  | t2 vs. t1 | -0,10 | [-0.5,0.3] | p=0.651 | Welch Two Sample t-test |

**Table S3:** Differences in media use between weekdays and weekends: At t0, media use was significantly higher at weekends. At t1, there was still a tendency of higher use at weekends, but the effects were considerably lower and did not reach level of significance.

| at t0 (before COVID-19) | | | | at t1 (shortly after lockdown) | | | |
| --- | --- | --- | --- | --- | --- | --- | --- |
| medium | OR weekend vs. weekday | confidence interval | p-value | medium | OR weekend vs. weekday | confidence interval | p-value |
| TV/video/DVD | 3,77 | [0.2,0.4] | p<0.001 | tv/dvd/streaming | 1,20 | [0.9,1.6] | p=0.222 |
| games console | 1,83 | [0.4,0.8] | p<0.001 | making videos | 1,08 | [0.8,1.5] | p=0.660 |
| computer/tablet | 1,33 | [0.6,1.0] | p=0.072 | computer games | 1,15 | [0.9,1.5] | p=0.337 |
| internet | 1,66 | [0.5,0.8] | p<0.001 | social media (active) | 1,00 | [0.7,1.3] | p=0.975 |
| mobile phone (without internet) | 1,03 | [0.7,1.3] | p=0.856 | social media (passive) | 1,17 | [0.9,1.6] | p=0.290 |
| mobile phone  (internet) | 1,44 | [0.5,0.9] | p=0.010 | reading books | 1,08 | [0.8,1.5] | p=0.594 |
| reading (electronic screen) | 1,44 | [0.5,1.0] | p=0.036 | reading magazins | 1,02 | [0.7,1.4] | p=0.912 |
| reading (paper) | 1,63 | [0.5,0.8] | p<0.001 | reading with parents | 1,15 | [0.8,1.7] | p=0.457 |
| music/radio play | 1,62 | [0.5,0.8] | p<0.001 | music/radio play | 1,03 | [0.8,1.4] | p=0.837 |
